# Supplementary material for: Live-cell imaging of glucose-induced metabolic coupling of β and α cell metabolism in health and type 2 diabetes
Source: Commun Biol. 2021 May 19;4:594. doi: 10.1038/s42003-021-02113-1 (PMC8134470; doi:10.1038/s42003-021-02113-1)
Supplement: Supplementary file 2 — Supplementary Information [file 42003_2021_2113_MOESM2_ESM.pdf]

Supplementary Information

Live cell imaging of glucose-induced metabolic coupling of β and α cell metabolism in health and type 2 diabetes

Zhongying Wang[1,2], Tatyana Gurlo[2], Aleksey V. Matveyenko[3], David Elashoff[4], Peiyu Wang[1,5,6], Madeline Rosenberger[2], Jason A. Junge[1,5], Raymond C. Stevens[1*], Kate L. White[1], Scott E. Fraser[1,5,6*], Peter C. Butler[2*].

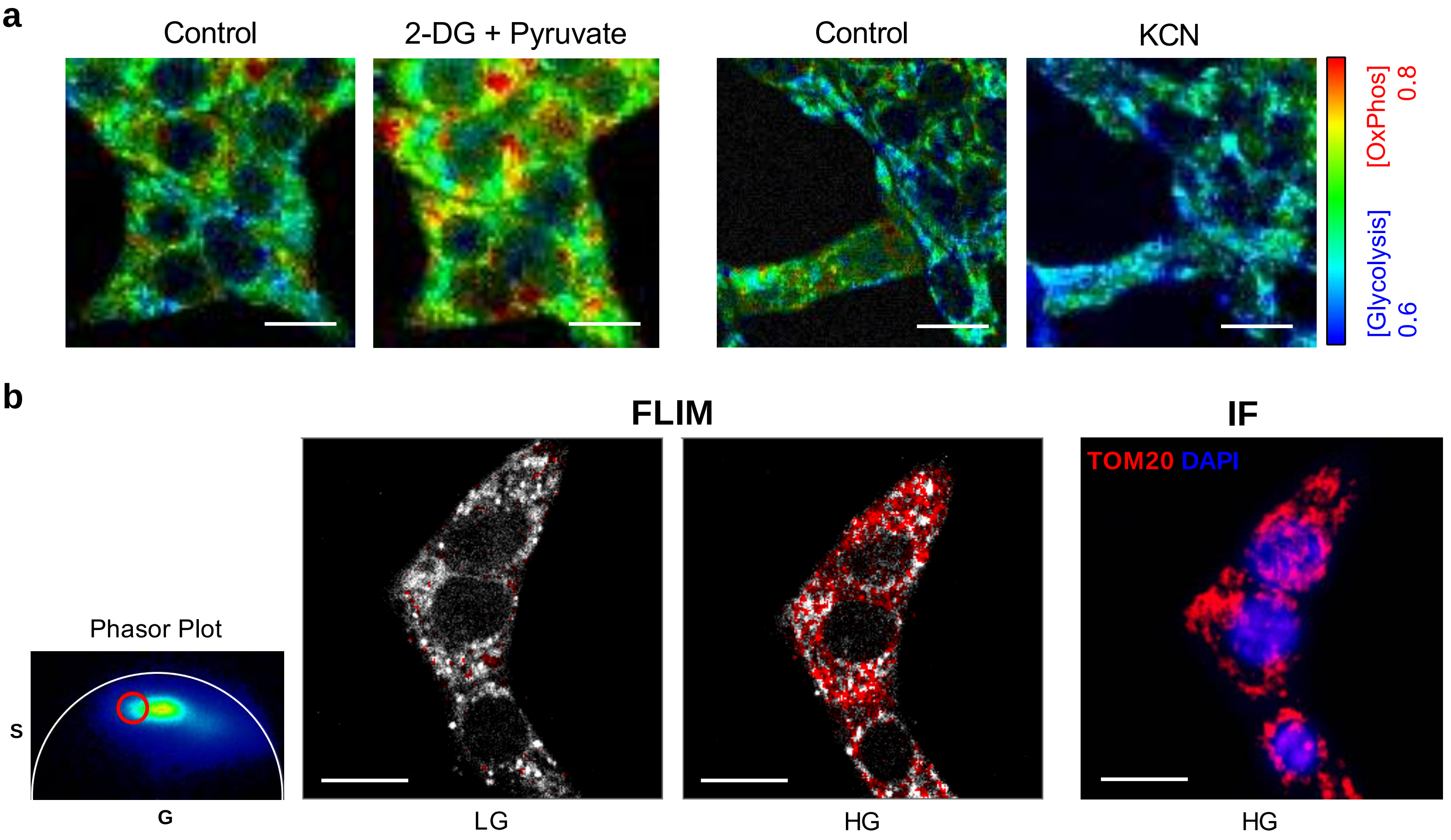

Supplementary Figure 1. FLIM signature in INS-1E β cells

(a) FLIM signature can be altered by blocking glycolysis with 15 mM 2-DG (2-Deoxy-D-glucose) and 1 mM pyruvate or blocking OxPhos with 4 mM potassium cyanide (KCN) in INS-1E β cells. (b) high glucose enhanced OxPhos with higher Bound/total NAD(P)H ratio (red masked according to phasor plot) in partial TOM20 (Santa Cruz Biotechnology sc-11415, Dallas, TX, USA, 1:200 for IF) positive mitochondrial area. Scale bar, 10 µm.

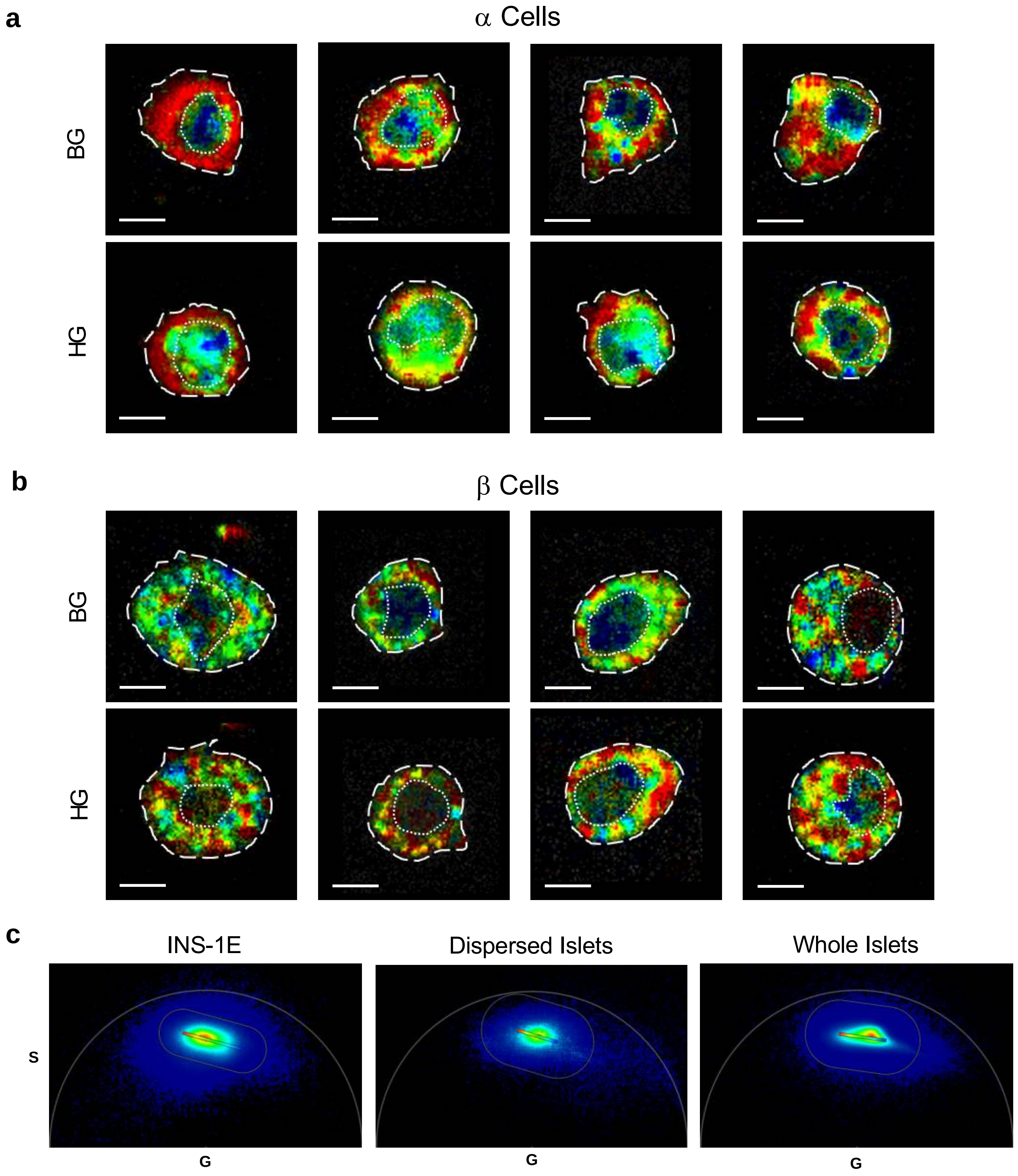

Supplementary Figure 2. FLIM signature of dispersed islet cells and phasor plot of INS-1E β cell, dispersed islets and whole islets

Dispersed islet cells illustrate subcellular OxPhos signal change. a, 4 representative dispersed β cells. High glucose enhanced OxPhos across the whole cell. b, 4 representative dispersed α cells. High glucose suppressed mitochondrial OxPhos close to the nucleus only. Scale bar, 5 µm. c, Overlapped phasor plot and rainbow mask of Figure 1, 2 and 3.

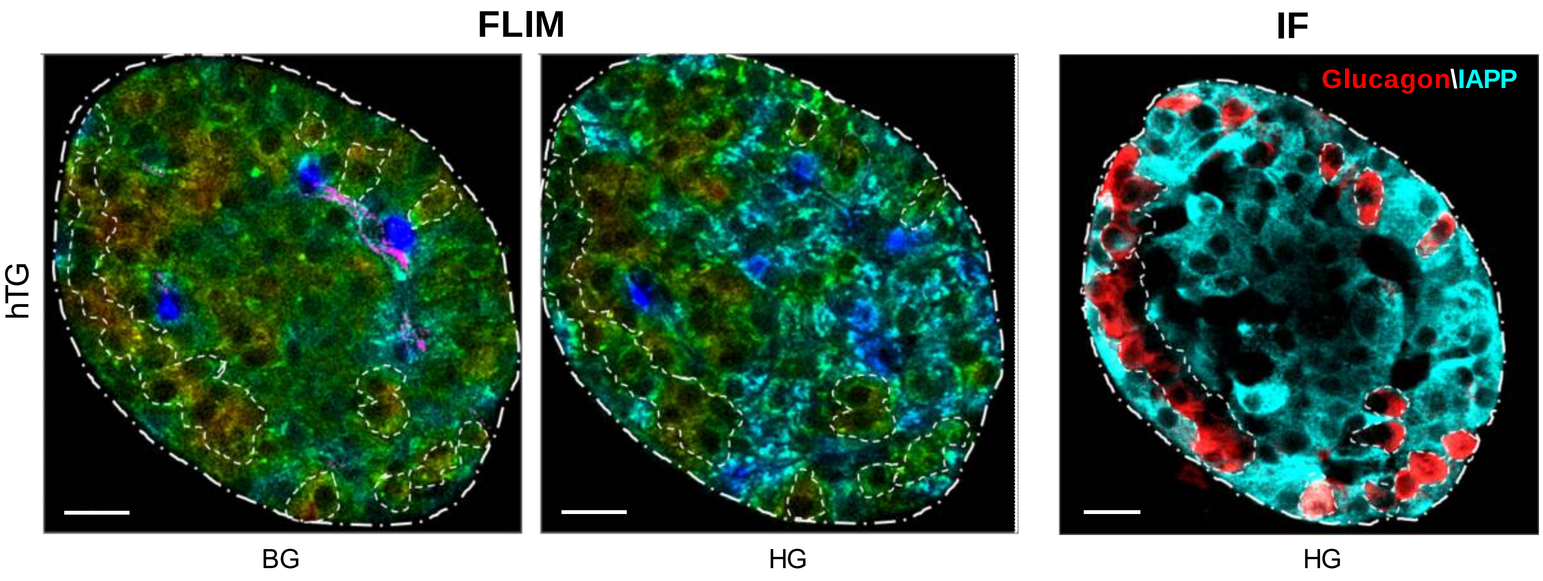

Supplementary Figure 3. Cell identity in hTG islets
hTG islet was imaged under basal glucose (BG, 4mM) and high glucose (HG, 16mM), following by immunofluorescent staining. α cells were stained by glucagon (red) and β cells by IAPP (cyan, Peninsula Laboratories Inc, San Carlos, CA, USA, 1:400 for IF). Scale bar, 20 μm.

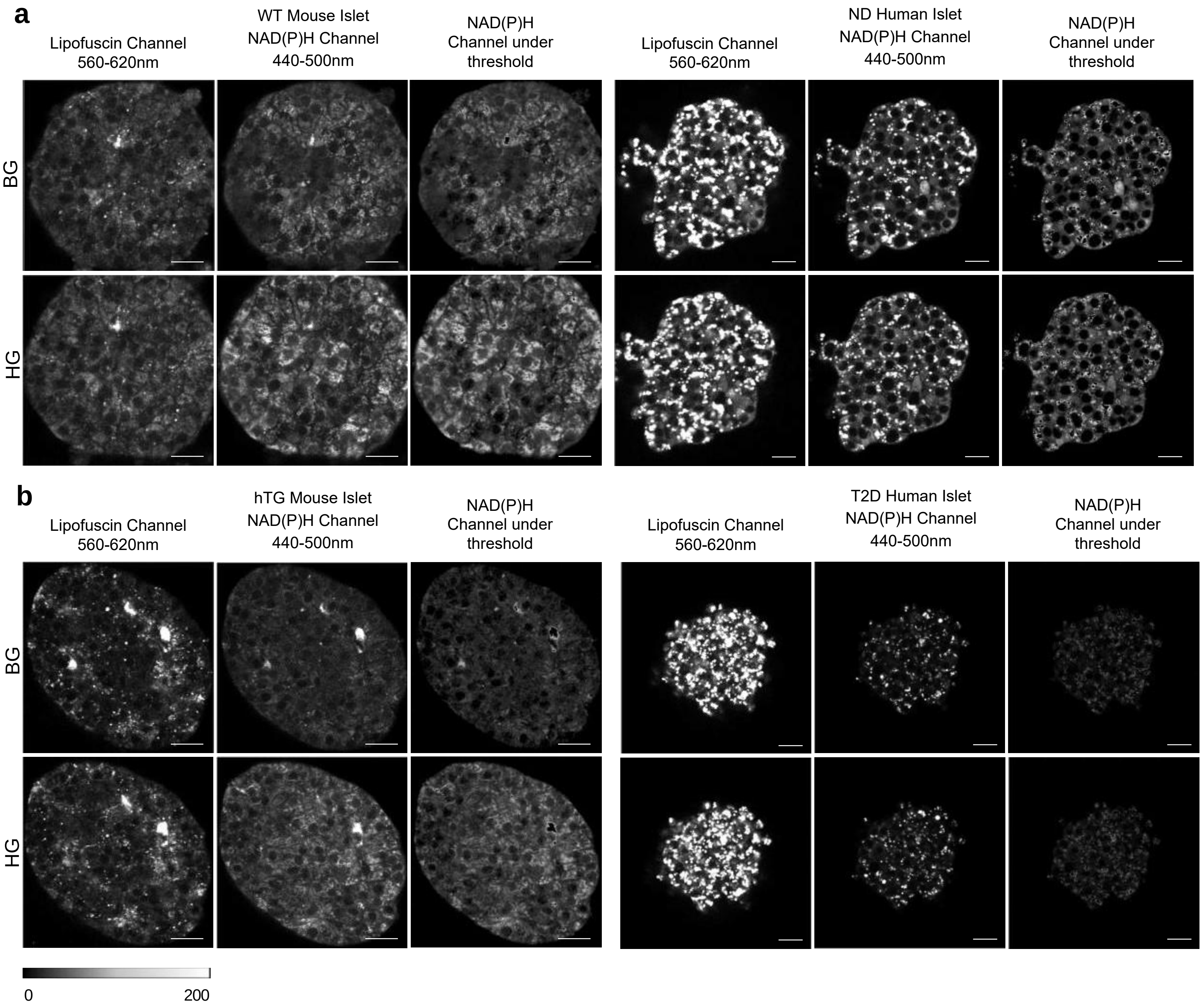

Supplementary Figure 4. Lipofuscin signal in rodent and human islets

Mouse islets had less lipofuscin (560-620 nm) than human islets (a and b). Human islets lipofuscin signal was prominent in the NAD(P)H channel (440-500 nm) under both basal and high glucose condition. By applying lower and upper threshold, pixels with lipofuscin can be removed in both sample types. Scale bar, 20 µm.

Supplementary Table 1. Characteristics of mice used for experiments.

Strain	Dispersed islets	Intact islets	
	WT	WT	hTG
n	3	6	4
BG	69.3±6.4	62.0±7.1	66.3±5.2
BW	21.6±1.7	26.1±0.5	24.4±0.8

BG – Blood Glucose; BW – Body Weight, Mean±SEM

Supplementary Table 2. Human islets used for FLIM imaging.

Donor	1	2	3	4	5
Source of islets	ProdoLabs	IIDP	IIDP	IIDP	IIDP
Unique identifier	HP-19102-01	SAMN 12227196	SAMN 15877725	SAMN 12597653	SAMN 12614852
Age (years)	40	51	31	42	65
Gender	F	M	M	F	F
Ethnic group	Hispanic	White	White	Hispanic	White
BMI (kg/m^2)	31.8	32.8	27.4	40.3	30.4
History of diabetes	ND	ND	ND	T2D	T2D
Cause of death	CVA	Cardiac arrest	Head trauma	CVA	CVA
Number of islets analyzed	1	2	3	4	4

ND – Non-diabetic; T2D – Type 2 Diabetes; IIDP - Integrated Islet Distribution Program; CVA - Cerebrovascular accident

Supplementary Table 3. Example of Bound/total NAD(P)H under different photon counts threshold.

	With lipofuscin pixels			W/o lipofuscin pixels		
	Photon count	Bound/total NAD(P)H	Pixel numbers	Photon count	Bound/total NAD(P)H	Pixel numbers
Mouse Islets	20-250	0.66	27003	20-100	0.66	24871
	20-400	0.66	27435	20-150	0.66	26665
Human Islets	20-400	0.68	22607	20-150	0.69	20672
	20-1000	0.68	23278	20-200	0.69	21894

Supplementary Table 4. Average Bound/total NAD(P)H ratio.

	INS-1E	Dispersed Islets		Intact Islets			
		WT α cells	WT β cells	WT α cells	WT β cells	hTG α cells	hTG β cells
LG/BG	0.655	0.708	0.643	0.661	0.651	0.661	0.640
HG	0.709	0.662	0.674	0.617	0.672	0.645	0.606
