# Supplementary material for: Live-cell imaging of glucose-induced metabolic coupling of β and α cell metabolism in health and type 2 diabetes
Source: Commun Biol. 2021 May 19;4:594. doi: 10.1038/s42003-021-02113-1 (PMC8134470; doi:10.1038/s42003-021-02113-1)
Supplement: Supplementary file 3 — Description of Additional Supplementary Files [file 42003_2021_2113_MOESM3_ESM.pdf]

Description of Additional Supplementary Files

File Name: Supplementary Data 1
Description: Data underlying plots shown in Figures 1-5
