# Supplementary material for: Live-cell imaging of glucose-induced metabolic coupling of β and α cell metabolism in health and type 2 diabetes
Source: Commun Biol. 2021 May 19;4:594. doi: 10.1038/s42003-021-02113-1 (PMC8134470; doi:10.1038/s42003-021-02113-1)
Supplement: Supplementary file 5 — Reporting Summary [file 42003_2021_2113_MOESM5_ESM.pdf]

nature research

Corresponding author(s): Peter C. Butler
Raymond C. Stevens
Scott E. Fraser

Last updated by author(s): Apr 6, 2021

Reporting Summary

Nature Research wishes to improve the reproducibility of the work that we publish. This form provides structure for consistency and transparency in reporting. For further information on Nature Research policies, see our Editorial Policies and the Editorial Policy Checklist.

Statistics

For all statistical analyses, confirm that the following items are present in the figure legend, table legend, main text, or Methods section.

n/a	Confirmed	
☐	☒	The exact sample size (n) for each experimental group/condition, given as a discrete number and unit of measurement
☐	☒	A statement on whether measurements were taken from distinct samples or whether the same sample was measured repeatedly
☐	☒	The statistical test(s) used AND whether they are one- or two-sided<br>*Only common tests should be described solely by name; describe more complex techniques in the Methods section.*
☐	☒	A description of all covariates tested
☒	☐	A description of any assumptions or corrections, such as tests of normality and adjustment for multiple comparisons
☐	☒	A full description of the statistical parameters including central tendency (e.g. means) or other basic estimates (e.g. regression coefficient) AND variation (e.g. standard deviation) or associated estimates of uncertainty (e.g. confidence intervals)
☐	☒	For null hypothesis testing, the test statistic (e.g. F, t, r) with confidence intervals, effect sizes, degrees of freedom and P value noted<br>*Give P values as exact values whenever suitable.*
☒	☐	For Bayesian analysis, information on the choice of priors and Markov chain Monte Carlo settings
☐	☒	For hierarchical and complex designs, identification of the appropriate level for tests and full reporting of outcomes
☒	☐	Estimates of effect sizes (e.g. Cohen's d, Pearson's r), indicating how they were calculated

Our web collection on statistics for biologists contains articles on many of the points above.

Software and code

Policy information about availability of computer code

Data collection	LAS X (Version 3.5.5.19976)
Data analysis	LAS X FLIM/FCS (Version 3.5.6, Leica Microsystems, Inc, Buffalo Grove, IL), Matlab (Version R2019a, MathWorks, Inc, Natick, MA), and SAS V9.4 (SAS Institute, Cary, NC)

For manuscripts utilizing custom algorithms or software that are central to the research but not yet described in published literature, software must be made available to editors and reviewers. We strongly encourage code deposition in a community repository (e.g. GitHub). See the Nature Research guidelines for submitting code & software for further information.

Data

Policy information about availability of data

All manuscripts must include a data availability statement. This statement should provide the following information, where applicable:

- Accession codes, unique identifiers, or web links for publicly available datasets
- A list of figures that have associated raw data
- A description of any restrictions on data availability

The original data and code that support the findings of this study are available from the corresponding authors upon request.

Field-specific reporting

Life sciences study design

All studies must disclose on these points even when the disclosure is negative.

Sample size	INS-1E: sample size 6. Random view of cultured cells, with more than 10 cells in each field of view.<br>Mouse dispersed islets: sample size 8. All cells were tracked and successfully characterized by immunostaining.<br>Single cells analyzed in intact islets: sample size 47 for alpha cells and 72 for beta cells from 5 WT intact islets.<br>Mouse intact WT islets: sample size 11 for WT from 6 mice and 10 mice for hTG from 4 mice.<br>Human islets: sample size 6 for ND from 3 individuals and 8 for T2D from 2 individuals.<br>Rat: sample size 5 for WT rats and 11 for HIP rats.<br>Only males develop diabetes as with all the rodent models.
Data exclusions	One human islet was not included because of deformity.
Replication	Z.W. and T.G. independently evaluated the raw data.
Randomization	Not applicable. No treatments were applied.
Blinding	Not possible because the same cells/islets were analyzed before and after glucose application.

Reporting for specific materials, systems and methods

We require information from authors about some types of materials, experimental systems and methods used in many studies. Here, indicate whether each material, system or method listed is relevant to your study. If you are not sure if a list item applies to your research, read the appropriate section before selecting a response.

Materials & experimental systems

n/a	Involved in the study	
☐	☒	Antibodies
☐	☒	Eukaryotic cell lines
☒	☐	Palaeontology and archaeology
☐	☒	Animals and other organisms
☒	☐	Human research participants
☒	☐	Clinical data
☒	☐	Dual use research of concern

Methods

n/a	Involved in the study	
☒	☐	ChIP-seq
☒	☐	Flow cytometry
☒	☐	MRI-based neuroimaging

Antibodies

Antibodies used	Guinea pig anti-insulin (Abcam, Cambridge, MA, USA; Cat#195956, 1:400)<br>Mouse anti-glucagon (Sigma-Aldrich, St. Louis, MO, USA; Clone K79bB10, Cat#G2654, 1:1000)<br>Rabbit anti-amylin(Peninsula Laboratories Inc, San Carlos, CA, USA, 1:400)<br>Donkey anti-guinea pig F(ab)2 fragments conjugated to FITC<br>Donkey anti-mouse antibodies F(ab)2 fragments conjugated Alexa647<br>Donkey anti-rabbit antibodies F(ab)2 fragments conjugated to Cy3 (The Jackson Laboratories, West Grove, PA, USA)
Validation	All antibodies used were validated in house by positive and negative controls. There is also validation for each antibody on the manufacture's data sheet provided on the website. All antibodies are commercially available.

Eukaryotic cell lines

Policy information about cell lines

Cell line source(s)	INS-1E
Authentication	Kindly provided by Dr. Pierre Maechler, University Medical Center, Geneva, Switzerland
Mycoplasma contamination	Negative
Commonly misidentified lines (See ICLAC register)	Not applicable.

Animals and other organisms

Policy information about studies involving animals; ARRIVE guidelines recommended for reporting animal research

Laboratory animals	Mice, WT FVB and FVB-Tg(IAPP)-6Jdm/Tg(IAPP)6Jdm, Male, 9-10 wk.<br>Rat, Sprague-Dawley WT and HIP, Male , 6 month
Wild animals	None.
Field-collected samples	None.
Ethics oversight	The Research Safety & Animal Welfare Administration at UCLA (ARC #2004-119 and ARC#2004-114)

Note that full information on the approval of the study protocol must also be provided in the manuscript.
